# Editorial Notes

**Published:** 1934

**Authors:** 


### Editorial Notes

**Dinner to Professor Fawcett.**

On Saturday, 30th June, Professor Fawcett was entertained by his students past and present at the Clifton Down Hotel. They came from far and near to the number of over a hundred to mark their appreciation of his work as Professor of Anatomy and their indebtedness to his teaching. For forty-one years he has occupied the Chair of Anatomy in University College and the University.

At the conclusion of the Dinner a presentation was made to Mr. and Mrs. Fawcett. It took the form of a grandfather clock and a cheque. The speeches of V. B. Green-Armytage, S. V. Stock, Col. P. Mackie and A. L. Flemming bore eloquent testimony to the esteem and affection felt for him by many generations of Bristol Medical Students.

In his speech acknowledging the presentation Professor Fawcett gave a comprehensive survey of the School as he has known it during nearly half of its hundred years' existence.

His name will stand out as one of the greatest of teachers in the Bristol Medical School, and his services as Dean have marked him as an able administrator and a friendly counsellor. His interest in athletics, too, particularly as a cricketer, endeared him to his students.

**Honorary Degrees, Dr. G. Parker and Dr. P. Watson-Williams.**

AT the University Congregation held on the 30th June, 1934, two members of the medical profession in Bristol were the recipients of honorary degrees.

Dr. George Parker, who is Consulting Physician at the Bristol General Hospital, received the LL.D. degree. This was a tribute to Dr. Parker's eminence as a medical historian and also a well-deserved appreciation of his services for many years as Honorary Medical Librarian in the University. Dr. Parker has a long record of medical work in our city, and recognition of his professional standing came to him in 1924 when he was elected President of the Association of Physicians of Great Britain and Ireland. His researches in medical history have made him the fountain of knowledge as regards Bristol practitioners and institutions. His *History of Surgery in Great Britain* contained a great deal of new material about the licensing of practitioners outside of London, whilst his *History of the Bristol Medical School*, prepared for the Centenary of 1933, must be well known to all our readers.

On Dr. Patrick Watson-Williams, who for many years edited this *Journal*, was conferred an Honorary M.D. His distinctions have lain so completely in his professional work that the highest degree in medicine, given *honoris causa*, seemed the most fitting, although he already was an M.D. of the Universities of London and Bristol. As a pioneer in Laryngology and Rhinology, Dr. Watson-Williams takes an honourable place in the annals of medicine at large as well as in British medicine. Born in Bristol, educated at Clifton College, and an alumnus of the Bristol Medical School, he is worthily upholding the traditions of his forbears and connections.

**Royal Sanitary Institute, Health Congress.**

THE Health Congress of the Royal Sanitary Institute for 1934 was held in Bristol from 9th to 14th July. The President of the Congress was Dr. Stanley Badock, and his presidential address of welcome was admirable, both in its substance and the manner of its delivery. The local secretarial arrangements were in the hands of the Town Clerk (Mr. Josiah Green), the Medical Officer of Health (Professor R. H. Parry), and Dr. Walker Hall, with the assistance of Mr. C. W. M. Vincent. The City Treasurer (Mr. E. M. Tapson) acted as Honorary Local Treasurer. The Lord Mayor (Councillor F. C. Luke, J.P.) presided over one of the Sectional Conferences, that of representatives of Sanitary Authorities. The local secretaries of the various sections and conferences played no small part, under the guidance of the presidents and recording secretaries of sections, in providing most interesting discussions in comfortable surroundings. The receptions, visits and excursions were very highly appreciated. Bristol's buildings, industrial enterprise and glorious natural environment formed subjects of the warmest approbation by those who attended the Congress. The City and University Authorities co-operated most successfully in rendering the whole Congress memorable. On Friday the Minister of Health (Rt. Hon. Sir Hilton Young, G.B.E., D.S.O., D.S.C., M.P.) visited the Congress and delivered a public address on some aspects of the Housing Question, in which he paid some well-deserved compliments to our City Council for their housing efforts.

**Retirement of Professor R. S. Statham.**

THE decision of Professor Statham to resign the University Chair of Obstetrics and his appointment at the Royal Infirmary creates another serious gap in the ranks of Bristol's teachers and consultants. His return to work last year after his long illness was a matter of great satisfaction to his colleagues and patients, and his resignation has been received with general expressions of regret.

But it is quite certain that his wisdom and ripe experience will be fully appreciated in Cheddar, where he has gone to take up a partnership in the practice with which his uncle was identified for so many years. We may recall what Dr. John Brown said of Adams of Banchory: "No one could say that his learning lessened his readiness and his ability for the active duties of his calling in the full round of its requirements." Some of us might think it a step down to relinquish a Chair in the University for the rôle of a country practitioner, if it were not for that haunting line in Bernard Shaw's *Revolutionist's Hand-book*: "He who can, does: he who cannot, teaches."

All our good wishes go with Professor Statham, and we hope he will find health, happiness and activity in a part of the country where he is no stranger.

